# Store-operated calcium entry drives alcohol-exacerbated neuroinflammation in retinal degeneration

**DOI:** 10.1038/s41420-026-03074-2

**Published:** 2026-03-31

**Authors:** Théo Henrique de Lima-Vasconcellos, Bruna de Albuquerque Menezes, Marília Inês Móvio, Gabrieli Bovi dos Santos, Gabriela Maria Badin, William Silva, Alexandre Hiroaki Kihara

**Affiliations:** 1 Laboratório de Neurogenética, Universidade Federal do ABC, São Bernardo do Campo, SP Brasil; 2 Centro de Matemática, Computação e Cognição, Universidade Federal do ABC, São Bernardo do Campo, SP Brasil

**Keywords:** Microglia, Retinal diseases, Neuroimmunology, Inflammation, Mechanisms of disease

## Abstract

Patients with neurodegenerative diseases such as retinitis pigmentosa (RP) may engage in maladaptive coping behaviors, including alcohol misuse, which can aggravate disease progression. Neuroinflammation, a hallmark of RP, is largely driven by microglial activation and amplified when cells are primed by oxidative stress. Store-operated calcium entry (SOCE), primarily mediated by Orai1 channels, regulates microglial metabolism and inflammatory signaling. Here, we tested whether ethanol (EtOH) exacerbates RP-related neuroinflammation through SOCE-dependent mechanisms and whether 2-aminoethoxydiphenyl borate (2-APB) mitigates these effects. In mixed retinal cultures, a “double-hit” (oxidative stress + EtOH) triggered pronounced microglial activation, neuronal loss, and altered cytokine expression and correlation patterns, assessed by multiplex bead assays and hierarchical clustering analysis. Indeed, 2-APB restored ramified morphology and improved neuronal survival. Conditioned medium experiments revealed that both microglia and Müller cells responded to the double-hit, but only microglia were sensitive to SOCE inhibition. In vivo, subretinal delivery of EtOH and 2-APB in rd1 mice, a genetic model of RP, altered microglial morphology and reduced pro-inflammatory cytokine levels without affecting photoreceptor density. Notably, in situ assessment of CD86/CD206 showed no change in expression, indicating that microglial activation in vivo is better captured by morphological and cytokine-network alterations than by classical surface markers. These findings show that alcohol misuse amplifies retinal neuroinflammation in RP via calcium-dependent mechanisms and identify SOCE as a therapeutic target for limiting damage from systemic comorbidities in retinal degeneration.

## Introduction

Individuals with neurodegenerative disorders, such as retinitis pigmentosa (RP), frequently exhibit an elevated prevalence of neuropsychiatric comorbidities, including anxiety and depressive symptoms [1], which collectively contribute to a significant decline in the overall quality of life [2, 3]. These psychological stressors may act as secondary insults, in which maladaptive coping strategies, such as alcohol consumption, accelerate disease progression [4, 5]. Neuroinflammation plays a pivotal role in the onset and progression of various neurodegenerative and psychological disorders, including Alzheimer’s disease [6], Parkinson’s disease, and RP, and is considered one of the therapeutic targets [7, 8]. Microglia, the resident immune cells of the central nervous system (CNS), are key mediators of neuroinflammation, exhibiting remarkable plasticity, dynamically altering their morphology and molecular profile in response to stimuli, displaying a wide range of phenotypes [9, 10].

Their functional state is closely linked to their morphology, transitioning from a quiescent, highly ramified form to an activated, amoeboid state, enabling them to respond efficiently to microenvironmental changes. This morphological shift enhances mobility to sites of injury or infection, promotes efficient phagocytosis, and drives the release of cytokines, chemokines, and other inflammatory factors, shaping the inflammatory milieu [11]. In addition to their unique lineage, originating from erythromyeloid progenitors in the yolk sac [9, 10], microglia exhibit trained immunity, or “priming,” whereby prior exposure to inflammatory stimuli heightens their responsiveness to subsequent challenges [12]. This primed state is critical in neurodegenerative diseases, as these cells become hyperactivated with impaired function [13–15]. This phenomenon aligns with the “double-hit” (DH) effect, in which an initial insult (first hit), such as injury or infection, sensitizes the immune system, and a subsequent stressor (second hit) elicits an exaggerated inflammatory response [16, 17]. Intracellular Ca²⁺ is essential for microglial functions, including activation, phagocytosis, and cytokine production [18, 19]. Priming may involve toll-like receptor (TLR) activation and alterations in intracellular calcium (Ca²⁺) homeostasis [20]. TLRs are pattern recognition receptors expressed by microglia that detect pathogen- and damage-associated molecular patterns (PAMPs and DAMPs)[21]. Ethanol (EtOH) activates microglia through toll-like receptors (TLRs) and purinergic receptors, including the ionotropic P2X subtype. These pathways are closely linked to intracellular calcium dynamics [22]. Following cellular injury, extracellular purines such as ATP are released and act as danger signals. They trigger microglial responses, including migration toward injury sites and increased phagocytic activity. This signaling is directly related to store-operated calcium entry (SOCE) in microglia [23, 24].

Microglial Ca²⁺ influx is primarily mediated by SOCE, which is triggered by endoplasmic reticulum (ER) Ca²⁺ depletion [23–25]. STIM1/2 sensors and Orai1/2 channels coordinate this process and are essential for restoring ER Ca²⁺ levels via sarco/endoplasmic reticulum Ca²⁺-ATPases (SERCA) [26, 27]. Notably, 2-aminoethoxydiphenyl borate (2-APB) is a well-characterized SOCE inhibitor and serves as a valuable tool to study calcium-mediated microglial responses [26, 28, 29]. Here, we investigated whether EtOH exacerbates retinal neuroinflammation in a DH model involving prior oxidative stress. We focused on the role of intracellular Ca²⁺ signaling in this process, particularly SOCE, and assessed whether pharmacological inhibition with 2-APB could mitigate these effects. We employed both in vitro and in vivo approaches to determine microglial activation, glia-glia communication, and neuronal survival in the context of retinal degeneration.

## Results

### 2-APB attenuates EtOH-enhanced microglial activation and neuronal loss in a double-hit in vitro model

**Fig. 1:**
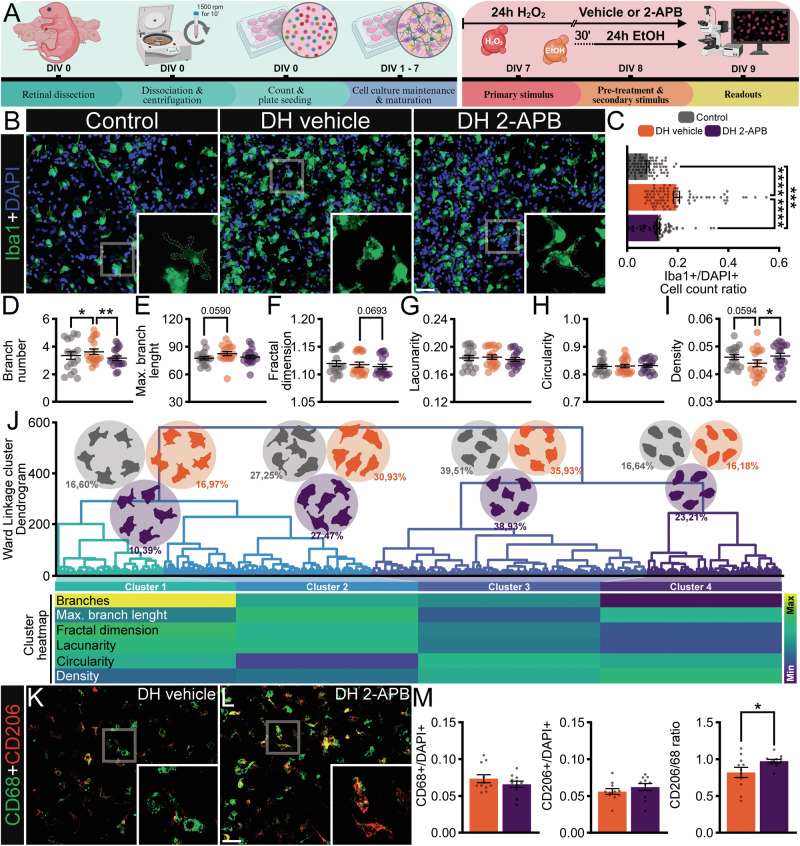
In vitro double-hit in retinal cells: effects of 2-APB on microglial inflammatory response. **A** Illustration of the experimental timeline applied to our double-hit model. **B** Representative immunofluorescence of ionized calcium-binding adapter molecule 1 (Iba1, green – microglia), counterstained with 4′,6-diamidino-2-phenylindole (DAPI, blue – nucleus). **C** Ratio of the number of Iba1-positive cells per DAPI-labeled nucleus, *n* = 10–13 wells. **D**–**I** Analysis of morphological parameters of microglia isolated from each group, considering (**E**) branch number, **F** maximum branch length, **G** fractal dimension, **H** lacunarity, **I** circularity, and **J** density, 3000–6500 individual microglia analyzed. **B**–**J** Experiments *n* = 6–9 technical replicates. **J** Hierarchical clustering dendrogram using Ward linkage of microglial cells sampled from each group, the ordinate corresponds to the linkage distance measured based on 21 features, defining 4 distinct morphological clusters determined by the elbow method following Thorndike’s procedure, which were color-coded gray, purple, and orange. The percentage of cells is represented by the total number of cells divided by the number of cells in each cluster. **K**, **L** Representative immunofluorescences of cluster differentiation 68 (CD68, green) and cluster differentiation 206 (CD206, red). **M** Left to right: Ratio of the number of CD68-positive cells per DAPI-labeled nuclei, ratio of the number of CD206-positive cells per DAPI-labeled nuclei, ratio of the number of CD206-positive cells per CD68-positive cells, *n* = 12 technical replicates. DIV = days in vitro. Values indicate mean ± SEM. **C**, **M** Bars represent standard errors of the means. **D**–**I** Scatter dot-plot represents mean ± SEM. The values that compose the mean are expressed as black dots in both graphs, *P < 0.05, **P < 0.01, ***P < 0.001, ****P < 0.0001, **C**–**I** Mixed-effects followed by Two-stage step-up method of Benjamini, Krieger and Yekutieli post-hoc and **M** Unpaired Student’s *T* test. Scale bar: 50 μm. Illustration created with Biorender.com.

We initiated our study using an in vitro model of mixed primary retinal cultures stressed with a DH strategy, as illustrated in Fig. 1A. The aim was to evaluate the effects of ethanol (EtOH) consumption on retinal cells already undergoing oxidative stress, mimicking the pathophysiology of RP. Briefly, we used H_2_O_2_ as the “first-hit”, a direct inducer of oxidative stress, which was applied for 24 h at a concentration previously used in our laboratory (50 µM), causing a milder decrease in cell viability and neuroinflammatory response [30]. After the incubation period, the vehicle or 2-APB was added to the wells and incubated for 30 min before the addition of the “second-hit” 50 mM EtOH, maintained for 24 h (Fig. 1A). The EtOH concentration was chosen based on the literature to reflect clinically relevant blood alcohol levels and commonly used in vitro exposures. We used concentrations within the range reported in alcohol-dependent individuals (~50–125 mM) and maintained exposure for 24 h in microglial and neuronal cell cultures, consistent with previous studies [22, 31, 32].

The concentration of 2-APB was determined using the MTT assay, and we chose 25 µM for further experimentation (Supplementary Fig. 1A). Exposure to H₂O₂ and the DH model significantly reduced cell viability (>70%), whereas EtOH had no effect. The PrestoBlue assay did not detect this reduction (Supplementary Fig. 1B, C). After establishing the DH model and determining the appropriate concentration of 2-APB, we aimed to examine microglial morphology and phenotype (Fig. 1B–M). The number of Iba1-positive cells serves as an indicator of microglia presence and activation, while morphological parameters provide insight into the state and activation phenotype of the microglia [11]. The Iba1 + /DAPI+ ratio revealed a significant increase in Iba1-positive cells in the single stimulus and DH approaches. In contrast, pre-treatment with 2-APB significantly reduced the number of Iba1-positive cells in the DH model, which remained higher than in the control (Fig. 1C and Supplementary Table S2–3).

Morphological analysis showed that the DH model intensified microglial changes, while a single stimulus had less impact (Supplementary Fig. 1E–J and Supplementary Table S2). DH resulted in a less complex morphology, but 2-APB pre-treatment mitigated the effects of the second hit (Fig. 1D–I and Supplementary Table S3). Four main clusters were observed in dendrogram analysis, revealing marked changes in all clusters. The morphological shift between DH 2-APB and the control was more pronounced than with the DH vehicle, indicating greater microglial activation with 2-APB (Fig. 1J). Phenotypic analysis with cluster of differentiation 68 (CD68, activated) and cluster of differentiation 206 (CD206, anti-inflammatory) revealed a significantly increased CD206/CD68 ratio in the DH 2-APB group, suggesting a shift toward an anti-inflammatory microglial phenotype (Fig. 1K, M).

**Fig. 2:**
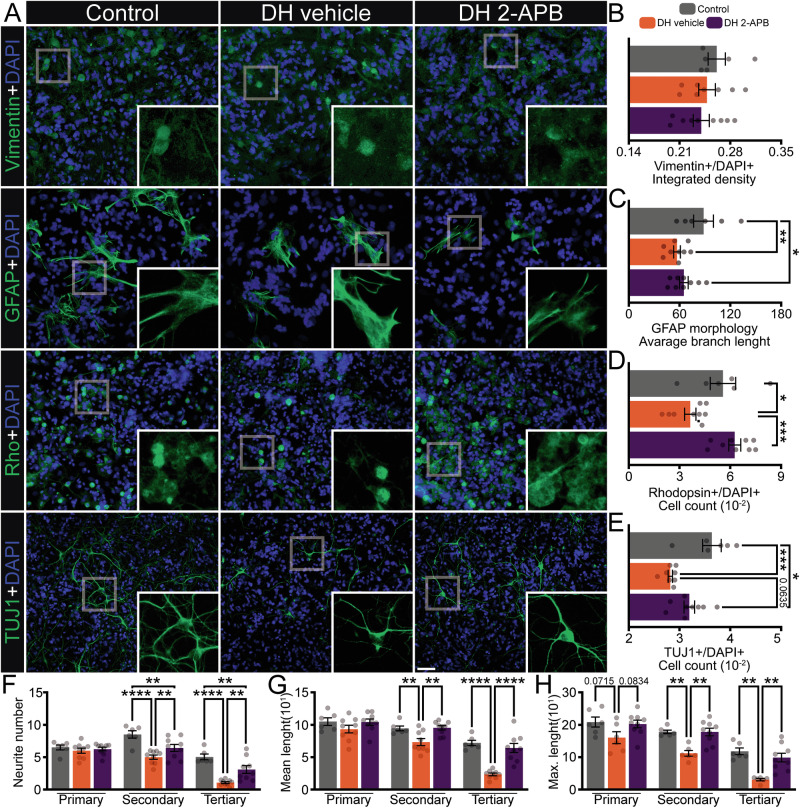
Effects of 2-APB in macroglial cells and neurons submitted to double-hit in retinal cell cultures. **A** Representative immunofluorescence of vimentin (green, Müller), glial fibrillary acid protein (GFAP, green – astrocyte), rhodopsin (Rho, green – rod photoreceptor), and class III beta-tubulin (TUJ1, green – neuronal cells), counterstained with 4′,6-diamidino-2-phenylindole (DAPI, blue – nucleus), with digital magnification in different conditions: control, DH pre-treated with vehicle, and DH pre-treated with 2-APB, respectively. **B** Integrated density of vimentin per DAPI, *n* = 6–9 wells. **C** Analysis of morphological parameters of astrocytes, considering the average branch length, *n* = 6–9 wells. **D** Ratio of the number of Rho-positive cells per DAPI-labeled nuclei, *n* = 6–9 wells. **E** Ratio of the number of TUJ1-positive cells per DAPI-labeled nuclei, *n* = 6–9 wells. TUJ1 neurite analysis: **F** number, **G** mean neurite length, and **H** maximum neurite length, *n* = 130–220 cells from 6–9 wells. Experiments *n* = 3 biological replicates × 2–6 technical replicates. Values indicate mean ± SEM. **B**–**H** Scatter dot plot with bars represents mean ± SEM. The values that compose the mean are expressed as black dots, *P < 0.05, **P < 0.01, ***P < 0.001, ****P < 0.0001. In (**B**, **D**, **E**), one-way ANOVA followed by Bonferroni’s post-hoc. **C** Mixed-effects followed by the Two-stage step-up method of Benjamini, Krieger, and Yekutieli post-hoc, and (**F**–**L**) two-way ANOVA followed by Bonferroni’s post-hoc. Scale bar: 50 μm.

Macroglial cells and neurons were analyzed to assess the broader effects of 2-APB on neuroinflammation and neuroprotection within the established DH model (Fig. 2A). Vimentin integrated density analysis revealed Müller cells in the DH vehicle group did not alter (Fig. 2B). Astrocyte analysis showed a reduction in average branch length (Fig. 2C and Supplementary Table S4). These results suggest that the DH model also induces macroglial alterations, albeit to a lesser extent than microglial cells.

Given these neuroinflammatory effects, subsequent experiments evaluated neuronal survival and morphology, providing insights into the neuroprotective potential of 2-APB (Fig. 2A). The DH vehicle group showed a significant decrease in Rho+/DAPI+ and TUJ1+/DAPI+ ratios compared to the control. At the same time, 2-APB pre-treatment increased both ratios, slightly surpassing control levels (Fig. 2D, E). The morphology of neuronal cells was analyzed using the NeuroJ plugin in ImageJ. Neurites were classified according to their branching order: primary neurites were defined as the processes emerging directly from the neuronal soma; secondary neurites were those branching from primary neurites; and tertiary neurites were defined as branches originating from secondary neurites. Both DH groups exhibited a significant reduction in secondary and tertiary neurites compared to the control group. However, mean and maximum neurite lengths were only reduced in the DH vehicle group. Compared to the DH groups, 2-APB pre-treatment partially reduced the impact of the second hit on neurite number, mean length, and maximum length (Fig. 2F–H and Supplementary Table S5). The DH model triggers neuroinflammation and neuronal loss, ultimately affecting both glial and neuronal morphology. Pre-treatment with 2-APB attenuates these detrimental effects, likely by modulating the neuroinflammatory response and preserving neuronal complexity under stress conditions.

### Conditioned medium implicates microglia as mediators of EtOH-induced neurotoxicity and 2-APB protection

**Fig. 3:**
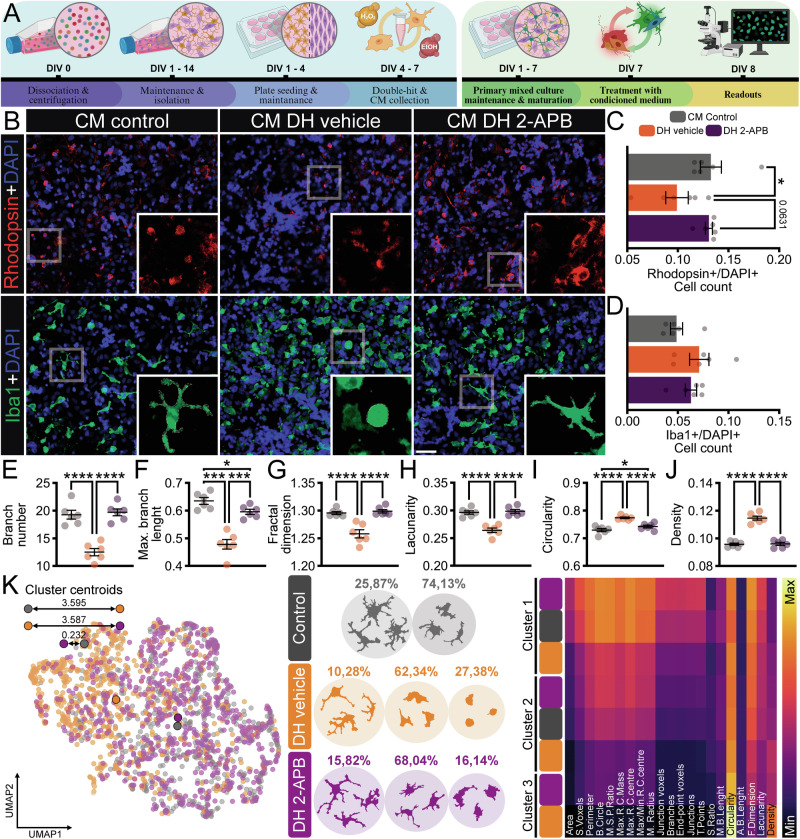
Effects of conditioned medium from pure microglia cells under a double-hit model in retinal cells. **A** Illustration of the experimental timeline used for obtaining the conditioned medium (CM) from pure glial cells to apply to primary mixed retinal cells. **B** Representative immunofluorescence images of rhodopsin (Rho, red - rod photoreceptor) and ionized calcium-binding adapter molecule 1 (Iba1, green - microglia), counterstained with 4′,6-diamidino-2-phenylindole (DAPI, blue – nucleus), with digital magnification under different conditions: control, DH pre-treated with vehicle, and DH pre-treated with 2-APB, respectively. **C** Ratio of the number of Rho-positive cells to DAPI-labeled nuclei, *n* = 5–6 wells. **D** Ratio of the number of Iba1-positive cells to DAPI-labeled nuclei, *n* = 5–6 wells. Analysis of morphological parameters of microglia isolated from each group, considering (**E**) branch number, **F** maximum branch length, **G** fractal dimension, **H** lacunarity, **I** circularity, and **J** density, *n* = 599–676 individual microglia from 5 to 6 wells. **K** From left to right: Uniform Manifold Approximation and Projection (UMAP) plot of the 21 features of microglia from the three groups. Representative cells from 2 to 3 distinct morphological clusters defined by a hierarchical clustering dendrogram using Ward linkage of microglial cells sampled from each group, based on 21 features. The percentage of cells is represented by the total number of cells divided by the number of cells in each cluster. Heatmap depicting the average value of each parameter of the cells shown in each cluster. Experiments included *n* = 2 biological replicates × 5–6 technical replicates. Values indicate mean ± SEM. **C**, **D** Bars represent standard errors of the means. **E**–**J** The scatter dot plot represents mean ± SEM. The values that compose the mean are expressed as black dots in both graphs, *P < 0.05, ***P < 0.001, ****P < 0.0001, in Mixed-effects followed by Two-stage step-up method of Benjamini, Krieger, and Yekutieli post-hoc. Scale bar: 50 μm. Illustration created with Biorender.com.

**Fig. 4:**
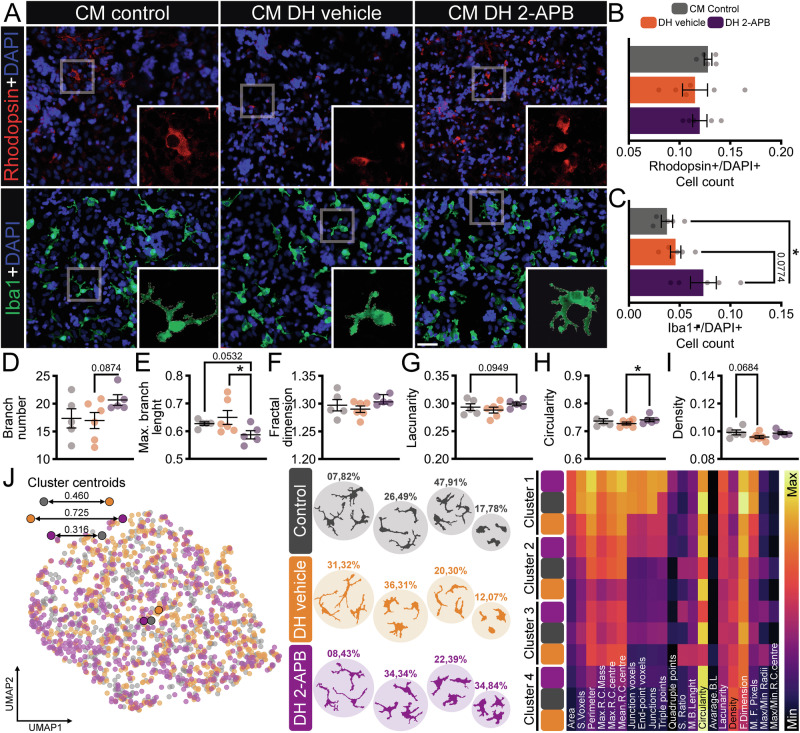
Effects of conditioned medium of pure Müller cells under the double-hit model in retinal cells. **A** Representative immunofluorescence of rhodopsin (Rho, red - rod photoreceptor) and ionized calcium-binding adapter molecule 1 (Iba1, green - microglia), counterstained with 4′,6-diamidino-2-phenylindole (DAPI, blue – nucleus), with digital magnification in different conditions: control, DH pre-treated with vehicle, and DH pre-treated with 2-APB, respectively. **B** Ratio of the number of Rho-positive cells per DAPI-labeled nuclei, *n* = 5–6 wells. **C** Ratio of the number of Iba1-positive cells per DAPI-labeled nuclei, *n* = 5–6 wells. Analysis of morphological parameters of microglia isolated from each group, considering **D** branch number, **E** maximum branch length, **F** fractal dimension, **G** lacunarity, **H** circularity, and **I** density, *n* = 550–600 individual microglia from 5 to 6 wells. **J** From left to right: Uniform Manifold Approximation and Projection (UMAP) plot of the 21 features of the microglia from the three groups. Representative cells from 2 to 3 distinct morphological clusters defined by a hierarchical clustering dendrogram using Ward linkage of microglial cells sampled from each group based on 21 features. The percentage of cells is represented by the total number of cells divided by the number of cells in each cluster. A heatmap depicts the average value of each parameter of the cells presented in each cluster. Experiments: *n* = 2 biological replicates × 5–6 technical replicates. Values indicate mean ± SEM. **E**–**J** The scatter dot plot represents mean ± SEM. **C**, **D** Bars represent standard errors of the means. The values that compose the mean are expressed as black dots in both graphs. *P < 0.05, **P < 0.01, ***P < 0.001, ****P < 0.0001, in Mixed-effects followed by Two-stage step-up method of Benjamini, Krieger and Yekutieli post-hoc. Scale bar: 50 μm.

To further investigate the cellular origin of the inflammatory response and determine whether the 2-APB-mediated protection observed in vitro was associated with modulation of the microenvironment, we designed a conditioned medium (CM) transfer experiment (Fig. 3A). We applied the DH model to primary pure microglial or Müller cell cultures; the CM was collected and used in primary mixed retinal cultures (Fig. 3A and Supplementary Fig. 2). We analyzed photoreceptor and microglial cells using this experimental setup (Figs. 3B and 4A).

The CM from microglia cells exposed to the DH vehicle model led to a substantial decrease in the Rho+/DAPI+ ratio and an increase in the Iba1+/DAPI+ ratio compared to the CM control (Fig. 3C, D). In contrast, the CM from microglia pre-treated with 2-APB resulted in a higher number of Rho+ cells, remaining similar to the CM control (*P* > 0.05), while Iba1+ levels remained elevated (Fig. 3D). Morphological analysis of microglia revealed a similar pattern of the DH model: CM DH vehicle enhanced the amoeboid/activated state in the microglial population, while CM DH 2-APB ameliorated this effect (Fig. 3K). This was reflected in several parameters (Fig. 3E–J and Supplementary Table S6). These results were evident in the cluster analysis, which showed a very similar pattern between the CM control and CM DH 2-APB clusters. Meanwhile, the CM DH vehicle displayed a distant cluster in the UMAP analysis. In addition, the heat map details the parameters of the cells present in each cluster, demonstrating that the CM DH 2-APB group is very similar to the CM control. At the same time, the CM DH vehicle becomes even more disparate (Fig. 3K).

The CM from Müller cells displayed intricate results: the group that received Müller CM from DH vehicle exhibited a decrease in the Rho + /DAPI+ ratio, but this was less impactful than CM from microglial cells, while CM 2-APB remained similar to the CM control (Fig. 4B). The Iba1+/DAPI+ ratio demonstrated a significant increase in both groups that received CM from Müller exposed to the DH model compared to the CM control group, with a greater increase observed in the pre-treatment with 2-APB (Fig. 4C). Morphological analysis revealed a similar pattern in microglial morphology—microglia receiving the CM from DH vehicle were less ramified, whereas exposure to Müller CM from DH 2-APB influenced microglia to adopt a more ramified and/or complex morphology, as indicated by skeleton analysis (Fig. 4D–I and Supplementary Table S7). When examining the main clusters by HCA, 4 clusters were identified in each group, with higher ramification in all 2-APB clusters, as depicted in the displayed cells and cluster heatmap (Fig. 4J).

We highlight that the DH model effectively mimics the compound stress conditions retinal cells might encounter in diseases like RP, providing a platform to study neuroinflammation and neurodegeneration. 2-APB demonstrated a protective role by reducing glial activation and promoting an anti-inflammatory phenotype, thereby preserving neuronal survival and maintaining neurite integrity. These findings underscore the potential therapeutic benefits of 2-APB in mitigating inflammation-related neurodegeneration in retinal diseases characterized by oxidative stress and external insults such as ethanol exposure. However, these observations were made in primary retinal cultures, which have limitations, including simplification of retinal structure and loss of the complexity of this layered tissue.

### In vivo administration of 2-APB modulates microglial morphology without rescuing photoreceptors

**Fig. 5:**
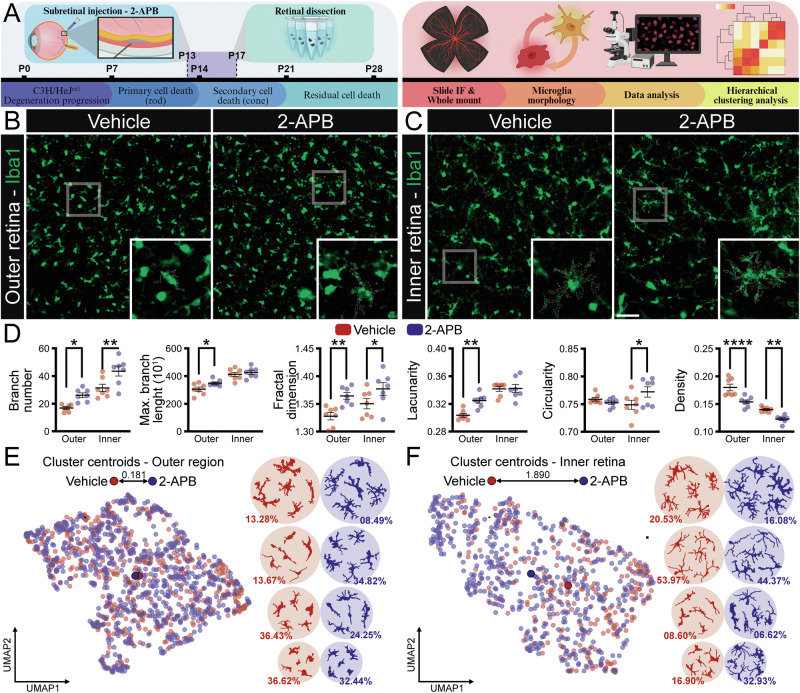
Pharmacological intervention with 2-APB in the arRP animal model and consequences for the neuroinflammatory response. **A** Illustration of the experimental timeline applied for subretinal injection and retinal analysis. **B**, **C** Representative immunofluorescence images of ionized calcium-binding adapter molecule 1 (Iba1, green – microglia), counterstained with 4′,6-diamidino-2-phenylindole (DAPI, blue – nucleus), from vehicle and 2-APB treatment injected via subretinal injection in C3H/HeJrd1 retinas 13 days after birth, collected at the age of 17 days in whole-mount, separated into outer (**B**, OPL-ONL) and inner retina (**C**, INL-IPL-GCL). **D** Analysis of morphological parameters of microglia isolated from Vehicle- and 2-APB-injected retinas, considering branch number, maximum branch length, fractal dimension, lacunarity, circularity, and density, separated into inner (INL-IPL-GCL) and outer (OPL-ONL) retina, *n* = 300–700 individual microglia from 7 animals. **E**, **F** From left to right: Uniform Manifold Approximation and Projection (UMAP) plot of the 21 features of the microglia from the three groups. Representative cells from four distinct morphological clusters were defined by a hierarchical clustering dendrogram using Ward linkage of microglial cells sampled from each group, based on 21 features. The percentage of cells is represented as the total number of cells divided by the number of cells in each cluster, for the outer (**E**) and inner retina (**F**). **D** The scatter dot plot represents the mean ± SEM. The values that compose the average are expressed as dots. *P < 0.05, **P < 0.01, ***P < 0.001 in (D) Mixed-effects followed by Two-stage step-up method of Benjamini, Krieger, and Yekutieli post-hoc. The layers indicate the approximate localization of the ONL outer nuclear layer, OPL outer plexiform layer, IPL inner plexiform layer, INL inner nuclear layer, GCL ganglion cell layer. Scale bar: 50 μm. Illustration created with Biorender.com.

To evaluate the effects of 2-APB in vivo, we injected [1 mM] 2-APB diluted in absolute EtOH at postnatal day 13 and analyzed the consequences at P17, when we observed a higher number of microglial cells, just before the peak of rod cell death (Fig. 5A). To address potential effects of EtOH per se, we also tested an alternative vehicle (1% DMSO in PBS) in a subset of animals; no differences were observed between the EtOH vehicle and the DMSO vehicle or naïve eyes for the endpoints reported here (data now shown). Vertical retinal sections revealed that 2-APB did not preserve photoreceptor cells: the ONL thickness, rhodopsin marker, and TUNEL+ cell counts were not significantly different from those of the vehicle-treated group. We also assessed macroglial reactivity using GFAP, Nestin, and glutamine synthetase markers (in both astrocytes and Müller cells). None of these markers exhibited significant changes in vertical sections following 2-APB administration (Supplementary Fig. 3A–H).

Microglia in RP retinas, including in this model, exhibit significant alterations. Microglia are typically found in the inner (IPL) and outer (OPL) plexiform layers of healthy retinas. In degenerating retinas, they migrate towards the site of photoreceptor loss and undergo morphological and phenotypic changes [33, 34]. The Iba1+ analysis indicated a higher count of Iba1+ cells in the outer layers of the vehicle-treated retinas. Morphological analysis of microglia from the outer retina revealed greater complexity and size in the 2-APB group, with similar findings in the inner retina. Clustering analysis showed that 2-APB treatment increased heterogeneity in microglial morphology, with cells becoming more ramified and larger (Supplementary Fig. 3I–K and Supplementary Table S8).

**Fig. 6:**
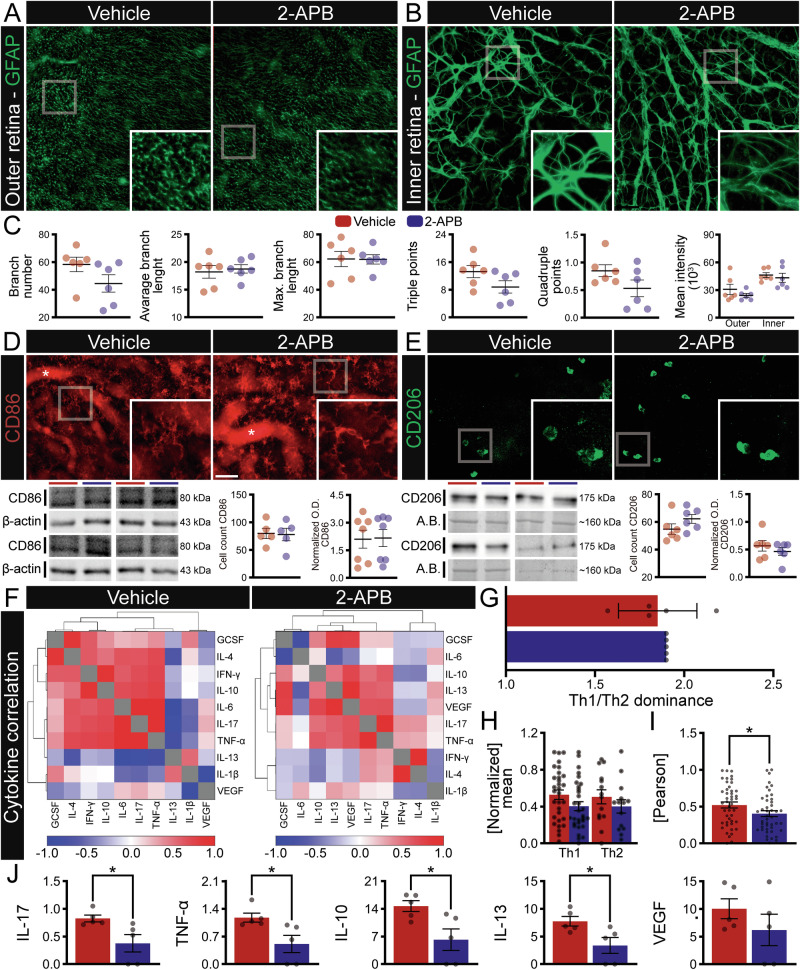
Integrated analysis of inflammatory cytokines in arRP mice after 2-APB subretinal injection. Representative immunofluorescence of glial fibrillary acidic protein (GFAP, green – astrocyte/Müller cells) from vehicle and 2-APB treatments injected via subretinal injection in C3H/HeJrd1 retinas 13 days after birth, collected at 17 days in whole-mount, separated into outer (**A**, OPL-ONL) and inner retina (**B**, INL-IPL-GCL). **C** Analysis of morphological parameters of GFAP whole photos from Vehicle- and 2-APB-injected retinas, considering branch number, average branch length, maximum branch length, triple points, quadruple points, and mean intensity, separated into inner (INL-IPL-GCL) and outer (OPL-ONL) retina, *n* = 7 animals. **D** Representative immunofluorescence cluster differentiation 86 (CD86, red – pro-inflammatory phenotype) and western blotting from vehicle and 2-APB treatments injected via subretinal injection in C3H/HeJrd1 retinas 13 days after birth, collected at 17 days in whole-mount, *n* = 5–7 animals. **E** Representative immunofluorescence cluster differentiation 206 (CD206, green – anti-inflammatory phenotype) and western blotting from vehicle and 2-APB treatments injected via subretinal injection in C3H/HeJrd1 retinas 13 days after birth, collected at 17 days in whole-mount, *n* = 5–7 animals. **F** Pearson’s correlation heatmap panel representing cytokines covariation, with the left side representing vehicle- and the right side 2-APB-injected retinas. Pearson’s correlation values range from −1.0 (blue) to 1.0 (red). **G** Th1/Th2 dominance in Vehicle- and 2-APB-treated retinas. **H** Normalized mean of Helper T cell type 1/pro- and 2/anti-inflammatory cytokines (Th1 and Th2). **I** Pearson’s correlation analysis in Vehicle- and 2-APB-treated retinas. **J** Graphs representing cytokine levels of IL-17, TNF-α, IL-10, IL-13, and VEGF for vehicle and 2-APB treatments injected via subretinal injection in C3H/HeJrd1 retinas 13 days after birth and collected at the age of 17 days. **C**–**E** The scatter dot plot represents the mean ± SEM. **E**–**H** Values indicate mean ± SEM. Bars represent standard errors of means. The values that compose the average are expressed in black dots. All cytokine analyses were performed using *n* = 5. *P < 0.05, in (**C**, **E**–**H**) Paired Student’s *T* test. Scale bar: 50 μm.

To complement the vertical analysis, we also performed Iba1+ and GFAP+ immunolabeling in whole-mount preparations, analyzing the inner (GCL, IPL, INL) and outer retinal layers (OPL, ONL) separately. This method enables a broader evaluation of the neuroinflammatory response by capturing a larger population of microglial cells and providing a more detailed visualization of macroglial structures, which tend to exhibit greater morphological complexity in whole-mount preparations (Figs. 5B, C and 6A, B). Iba1+ labeling in whole mounts revealed a pattern consistent with the vertical sections—2-APB increased microglial complexity in both retinal regions (Fig. 5D–F and Supplementary Table S9), with a higher number of Iba1+ cells counted in the inner retinal layers (Supplementary Table S9). Although the number of clusters remained similar, the treated group exhibited more ramified and morphologically complex microglia, particularly evident in the inner retinal layers (Fig. 5E, F and Supplementary Table S9).

### SOCE inhibition attenuates EtOH-induced proinflammatory cytokine expression in degenerating retinas

Interestingly, GFAP expression was unchanged, indicating that 2-APB modulates neuroinflammation primarily via microglial pathways rather than via gross Müller cell reactivity (Fig. 6A–C and Supplementary Table S10). Although we observed clear alterations in microglial morphology (Fig. 5), microglial phenotype as assessed by CD86 (pro-inflammatory) and CD206 (anti-inflammatory) immunolabeling remained unaltered in situ (Fig. 6E, F). By contrast, our in vitro experiments revealed both phenotypic shifts and changes in the secretome (Figs. 1–4), prompting us to profile retinal cytokines in vivo using a multiplex bead assay (Fig. 6D). Retinas treated with 2-APB exhibited reduced levels of IL-17, TNF-α, IL-10, and IL-13, whereas other mediators such as VEGF remained unchanged (Fig. 6H). Heatmap analysis showed stronger inter-cytokine correlations in vehicle-treated retinas (warmer colors) but a cooler, less coordinated cytokine network after 2-APB treatment, consistent with a lower mean Pearson correlation coefficient (Fig. 6F, G). Finally, analysis of Th1/Th2 balance revealed no significant shift following 2-APB administration (Fig. 6E–F).

## Discussion

### EtOH exacerbates oxidative stress–induced neuroinflammation: support for the “Double-Hit” hypothesis

Our findings reveal that short-term EtOH exposure intensifies neuroinflammatory responses and neural loss in retinal cells already primed by oxidative stress, mimicking conditions that may exacerbate neurodegeneration and supporting the “double-hit” hypothesis of glial sensitization. In our in vitro model, combining H₂O₂ and EtOH triggered a more robust inflammatory profile than either stimulus alone, characterized by microglial morphological activation, cytokine release, and neuronal degeneration. Pre-treatment with 2-APB, a well-known SOCE modulator, attenuated these responses, preserving neuronal integrity and decreasing microglial cell activation. In vivo, subretinal administration of 2-APB in a retinal degeneration model reshaped the glial inflammatory landscape, reinforcing the central role of SOCE in regulating microglial behavior during pathological stress. Although CD86 and CD206 are commonly used to distinguish pro- and anti-inflammatory microglial states, we observed no significant differences in their expression across experimental groups. This is consistent with evidence that microglial activation in vivo cannot be reduced to classical M1/M2 categories, as retinal microglia exhibit pronounced spatial and functional heterogeneity during both homeostasis and degeneration [33, 35]. Moreover, multiple conceptual reviews have emphasized that the traditional M1/M2 framework does not accurately represent microglial biology in the central nervous system, and that surface markers such as CD86 and CD206 are insufficient to define functional activation states [36]. Complementarily, analyses of M2-associated pathways indicate that microglia can adopt overlapping or transitional phenotypes, further challenging the validity of a binary polarization model and supporting the need for multidimensional readouts [37]. Accordingly, our in situ immunofluorescence and Western blot analyses indicate that morphological remodeling and changes in the cytokine/chemokine network are more sensitive indicators of microglial activation than CD86/CD206 alone. These results underscore the contribution of Ca²⁺ signaling in glial activation and suggest that modulating this pathway may reduce secondary damage in neurodegenerative conditions.

Previous studies from our group and others have shown that H₂O₂ concentrations of 50–100 µM can induce glial reactivity and cytotoxicity in primary retinal cultures, with higher concentrations eliciting amplified neuroinflammatory responses [30, 38]. Importantly, H₂O₂/oxidative stress also increases the expression of SOCE-related components, including Orai1, in retinal cells and glia [39]. EtOH, in turn, exacerbates microglial reactivity when applied to lipopolysaccharide (LPS) pre-exposed cells [40, 41]. In BV2 microglial cells, EtOH exposure (25–100 mM) increases expression of P2X4 and P2X7 receptors [41]—both key mediators of Ca²⁺ influx and glial activation. These effects have also been validated in primary microglia [41–43], and CM from EtOH-stimulated microglia induces neuronal degeneration and mitochondrial dysfunction in vitro [22, 44]. EtOH exposure induces microglial activation and morphological alterations in vitro, increasing IL-10 and TNF-α expression [31, 32, 44]. Collectively, these data support the interpretation that EtOH aggravates microglia that are already activated or sensitized.

Microglial priming is a recognized phenomenon in neurodegenerative diseases, in which prior inflammatory or oxidative stimuli sensitize microglia to secondary insults, leading to exaggerated responses, epigenetic modifications, and upregulation of markers such as CD68 [15, 45]. Models of spinal cord injury followed by systemic immune challenge have demonstrated distinct transcriptional priming signatures, consistent with the emergence of damage-associated microglial states [46]. Stimulation of P2X7 receptor by LPS in primary retinal microglia increases IL-1β expression and cytoplasmic Ca²⁺ levels, reinforcing the link between purinergic signaling and priming phenomena [47]. This is supported by evidence showing that knockdown of TRPC1, a SOCE component, reduces both SOCE amplitude and the priming response in microglia [48]. Our findings align with this literature, demonstrating that pre-exposure to oxidative stress enhances the microglial response to EtOH, and that 2-APB attenuates this exacerbated profile by targeting calcium influx mechanisms. We acknowledge that systemic alcohol exposure is clinically relevant and may produce broader neuroimmune changes; systemic models such as chronic oral gavage, vapor exposure, or Lieber–DeCarli diet would therefore be informative in future work. However, our aim in the present study was to probe retina-directed effects of EtOH and SOCE modulation while minimizing systemic confounders; local subretinal injection allows a spatially restricted perturbation and direct assessment of retinal microglial responses. We therefore used a local model here while acknowledging that translation to chronic systemic models is an important next step.

### Purinergic and TLR-mediated calcium signaling in microglial sensitization

Several studies have linked TLRs to intracellular calcium signaling through the IP3R/SOCE axis. In human mesenchymal stem cells, TLR3 and TLR4 priming enhance IP3R1-mediated Ca²⁺ responses and cytokine production [20]. Similarly, in macrophages, the TLR2–SOCE pathway is crucial for proinflammatory activation and bactericidal activity [49]. In microglia, IP3Rs sustain spontaneous Ca²⁺ transients, and their inhibition reduces activation [50]. Multiple studies utilized 2-APB in microglial cells [51–57]. These studies highlight that Orai1/CRAC channels, not TRPs, are the main mediators of SOCE in microglia, and that 2-APB modulates these channels in a dose-dependent manner, enhancing at low concentrations (1–5 μM) and blocking at higher concentrations (40–50 μM)[58]. Moreover, purinergic stimulation induces intracellular Ca²⁺ spikes in microglia, promoting TNF-α release; 2-APB blocks this effect in a dose-dependent manner (30–100 μM)[59]. P2Y13-mediated Ca²⁺ store depletion activates SOCE in spinal cord microglia, which is also inhibited by 2-APB [51]. These findings suggest that SOCE not only regulates Ca²⁺ homeostasis but also facilitates microglial communication via intercellular Ca²⁺ waves, as demonstrated in BV2 cells through P2Y12/13 receptor activation and ATP/ADP signaling—a process also inhibited by 2-APB [53].

SOCE supports calcium influx and orchestrates effector functions in microglia, including phagocytosis and cytokine release. The Orai1–STIM1 complex regulates these functions in murine primary microglia [60]. Pre-treatment with 2-APB reduces phagocytic activity following LPS or UDP stimulation, confirming the Ca²⁺ dependence of this process [60]. Likewise, genetic knockdown of STIM1, STIM2, or Orai1 impairs microglial migration and cytokine secretion, resulting in corresponding reductions in NF-κB signaling [61, 62].

These findings align with our observation that 2-APB attenuates microglial activation. Additional studies show that overexpression of selenoprotein K enhances microglial migration and phagocytosis by increasing cytosolic Ca²⁺ levels, which are suppressed by 2-APB [55]. CRAC-dependent Ca²⁺ influx is also essential for podosome formation, a prerequisite for microglial motility and invasion [63]. Furthermore, 2-APB downregulates cytokine expression—such as TNF-α, IL-1β, and IL-17—in both CNS tissue and cultured glial cells [59, 64]. Interestingly, Ca^2+^ profiles vary across microglial morphotypes: ramified microglia exhibit low basal Ca²⁺, while amoeboid cells display higher Ca²⁺ activity [65, 66]. These dynamic shifts may reflect functional transitions modulated by SOCE and potentially be targetable for regulating microglial states. In line with this, helminth causing neurocysticercosis produces soluble factors that inhibit TRPC1 and Orai1 Ca^2+^ channel-mediated activation of the NF-κB and MAPK pathways in microglia [67].

### Müller Glia–Microglia crosstalk and SOCE in retinal neuroinflammation

Glial crosstalk is well-known for its role in retinal homeostasis and neuroinflammation. Microglia-derived factors can induce Müller gliosis and promote the proliferation and generation of progenitor cells for regeneration [68]. Conversely, Müller glia-derived factors can modulate microglial activation and inflammatory tone, potentially limiting the extent of retinal damage [69]. In our study, CM assays suggest that Müller cells may contribute to dampening microglial-derived toxicity, although their response to 2-APB appears limited. While SOCE has been widely studied in astrocytes, its role in Müller glia remains poorly explored. Notably, in the rd10 model of retinal degeneration, Orai1 is upregulated in Müller cells exposed to oxidative stress, colocalizing with GFAP and enhancing SOCE currents [39]. Ex vivo 2-APB application in that model prevented neuronal death, suggesting SOCE involvement in Müller-mediated gliosis—though microglial responses and in vivo dynamics were not assessed in that study. Although numerous studies have explored the role of Ca²⁺ in RP, results remain inconclusive regarding the effectiveness of Ca²⁺-channel blockers or genetic ablation in preserving photoreceptors. While some studies suggest protective effects, others indicate that inhibiting Ca²⁺ channels either fails to reduce retinal cell death or may exacerbate degeneration, underscoring the complexity of Ca²⁺ dysregulation in these diseases [26, 70, 71].

In conclusion, our data demonstrate that EtOH exacerbates oxidative stress-induced neuroinflammation through calcium-dependent mechanisms, primarily by amplifying microglial activation, and that pharmacological inhibition with 2-APB attenuates these responses and helps preserve neuronal morphology. These findings position SOCE as a critical modulator of glial crosstalk and neurodegeneration in retinal diseases, highlighting its therapeutic potential, particularly for managing systemic comorbidities such as alcohol exposure. However, we acknowledge that this mechanistic interpretation remains tentative, as 2-APB exhibits well-known off-target actions. More definitive validation will require calcium-imaging assays (e.g., thapsigargin-evoked Ca²⁺ entry in the presence and absence of 2-APB), the use of more selective SOCE inhibitors, or genetic approaches such as Orai1/STIM1 knockdown or CRISPR-based manipulation. In addition, our study used local subretinal EtOH application to probe retina-directed effects and therefore does not substitute for systemic alcohol exposure models. While our findings implicate SOCE in EtOH-amplified microglial neuroinflammation and show 2-APB-mediated protection, the mechanistic conclusion remains provisional pending more selective pharmacological and genetic validation.

## Material and methods

### Ethics statement

These experiments were conducted in accordance with the guidelines of the NIH and the Brazilian Scientific Society for Laboratory Animals. Long Evans rats (Rattus norvegicus) were obtained from Biotério de Criação da UFABC (São Bernardo do Campo), RD1 (C3H/HeJrd1) was acquired from the Instituto de Ciências Biológicas (ICB) at the Universidade de São Paulo (USP), and C57BL/6 mice were sourced from the Biotério de Criação da UFABC (Santo André), using both sexes (males and females). The animals were maintained on a 12-hour light/dark cycle with water and food provided ad libitum, in an environment with controlled temperature and humidity (23-25 °C, relative humidity 75%). The experimental protocols were carried out with approval from the Ethics Committee of the Federal University of ABC (CEUA-UFABC #**5037101121**). The experimental protocols were carried out following the approval of the Ethics Committee of the Federal University of ABC (CEUA-UFABC #5037101121).

### Retinal primary mixed cell culture

P0-P4 neonates of Long Evans were euthanized by direct decapitation, retinas dissected and washed using DMEM medium before the retinas were digested with a 2.5% trypsin solution for 10 min at 37 °C (Thermo Fisher, #15090046). Fetal Bovine Serum (FBS, Corning, #35-010-CV) was added to deactivate the trypsin. The following steps were performed as previously described [30]. After precipitated homogenization with Neurobasal-A medium supplemented with 10% FBS, 1% Penicillin/Streptomycin antibiotic (P/S, 10 μg/ml, Gibco, #15070-063), 1% L-Glutamine (Gibco, #25030149), and 1% B27™ Supplement (50X, Gibco, #10889038). For oxidative stress experiments, B27-minus was employed once this reagent lacked antioxidants. The solution containing the retinal cells was distributed to wells that had been previously treated with poly-D-lysine (PDL, Gibco, #A3890401). Dissociated cells were counted and plated (3 × 10^4^/well in 96 well plates and 12 × 10^4^/well in 48 well plates, 18 × 104/well in 24 well plates), and the cells were kept in an incubator with 5% CO_2_, 37 °C, controlled humidity until reaching 7 days in vitro (DIV). The cell culture media were changed every 2–3 days.

### Retinal primary mixed glial cell culture

This method was adapted from [72]. P0-P4 neonates of Long Evans were euthanized by direct decapitation, retinas dissected and washed using DMEM medium before the chemical digestion. Cells were cultured in 75 cm² flasks using DMEM/F12 medium (Gibco, #12500-062) supplemented with 10% FBS, 1% P/S, and GlutaMAX (Gibco, #35050-061), maintained at 37 °C with 5% CO_2_. The culture medium was renewed after one week to remove cell debris. After 14 days of culture, the microglia were separated/isolated from Müller cells using an orbital shaking technique at 120 rpm for 1 h at 37°C. Then, the medium with the microglia cells was collected and centrifuged for 5 minutes at 1000 rpm; the supernatant was discarded, and the pellet, containing microglia, was resuspended in the medium. The Müller cells were detached from the flask using trypsin/EDTA (Gibco, #25200056) for 5 min, then centrifuged for 5 min at 1000 rpm. Once isolated, the microglia and Müller cells were plated separately at a density of 10^4^ cells in 48-well plates pretreated with PDL and used for treatments after 4 days in culture. To confirm the purity obtained by this technique, immunofluorescence labeling of anti-Iba1 and -Vimentin was used. The positive cells were manually counted using ImageJ software, and a purity of greater than 95% was achieved (Supplementary Fig. 2).

### The double-hit model (DH)

After 7 DIV of primary mixed cell cultures, the medium was exchanged by adding 50 µM hydrogen peroxide (H_2_O_2)_ for 24 h [30]. After this incubation period, 25 µM of 2-APB (Sigma, #D9754) or the same volume the vehicle (dimethyl sulfoxide (DMSO) in phosphate-buffered saline (PBS)) was applied 30 min before the second stimulus, 50 mM ethanol (0.3% v/v) for 24 h (See Fig. 1A for illustrated protocol). After 24 h, cells were fixed with 4% paraformaldehyde (PFA). The medium was collected from pure cultures for further experimentation.

### MTT tetrazolium reduction assay

The cellular viability was determined by the reduction test of 1-(4,5-dimetiltiazol-2-il)-3,5-difenilformazan assay (MTT, Sigma-Aldrich, #M2128). All treatments were performed for 24 h in 96-well plate and 5% CO_2_. After the incubation period, fresh medium was replaced with cell medium, and 10 μL of MTT solution (5 mg/mL) was added. After 2 h of incubation, the cell medium containing the MTT solution was replaced with pure DMSO (Synth, Brazil, #D1011.01.BJ) without dilution. The absorbance values were obtained in an ELISA plate reader (SpectraMax M5) at 570 nm (reference at 620 nm). Data were normalized to values obtained from untreated wells, and experiments were performed at least 3 times.

### PrestoBlue resazurin reduction assay

Cellular viability was determined using the resazurin reduction test (Thermo Scientific, #A13261). All treatments were performed for 24 h in 96-well plate and 5% CO_2_. After the incubation period, 1/10 volume of PrestoBlue solution was added and incubated for 2 hours. The absorbance was measured using a SpectraMZX M5 spectrophotometer at 570 nm and 600 nm wavelengths, with a reference wavelength of 620 nm. The excitation wavelengths of 560 nm and 590 nm, and the corresponding emission wavelengths, were used to read fluorescence. Data were normalized to values from untreated wells, and at least 3 independent experiments were performed.

### Conditioned medium treatment

To analyze the secretomes of microglia and Müller cells separately, we collected the supernatant (medium) from primary pure cultures post-double-hit induction. The collected conditioned medium (CM) was centrifuged for 5 min at 900 rpm to remove debris and other particles. The CM was stored at −80 °C until use. The CM was applied by replacing 1/3 of the media in retinal mixed primary cultures at 7 DIV. After 24 h, cells were fixed with 4% paraformaldehyde (PFA).

### Subretinal injection

2-APB (Sigma, #D9754) was prepared at a concentration of 1 mM in ethanol P.A.; 1 µL of this solution was injected into the subretinal space of the right eye using the trans-scleral subretinal injection method [70]. The same volume of vehicle (ethanol P.A.) was injected into the left eye as a control. C3H/HeJRD1 mice at postnatal day 13 (P13) were anesthetized with isoflurane (0.8–1.5 L/min at 3%) in an induction box and then placed on a sterile surface under a microscope. Anesthesia was maintained with isoflurane (0.8–1.5 L/min at 2%), and body temperature was kept at 37 °C using a heating pad. The loss of the pedal reflex confirmed the depth of anesthesia. The skin over the eyelid was disinfected, and a sterile 30-G needle was used to make a small incision along the future eyelid margin. The skin was gently displaced with sterile forceps to expose the eyeball. The drug or vehicle was then delivered into the subretinal space using a glass capillary needle, inserted tangentially to form a localized subretinal bleb under direct visualization. Successful bleb formation and absence of major complications (e.g., extensive retinal detachment or hemorrhage) were confirmed immediately after injection by high-magnification microscopy. The injection site was gently cleaned with sterile gauze and 0.9% NaCl. Animals were monitored for three days for signs of pain or complications and maintained under standard postoperative care. After four days, animals were euthanized with a lethal dose of urethane (1 g/kg), and retinas were dissected for downstream assays.

### Immunofluorescence (IF)

Immunofluorescence assays were performed on both in vitro and in vivo retinal sections. For in vivo experiments, the animals were anesthetized and euthanized. After decapitation, the eyes were dissected and post-fixed in 4% PFA in 0.1 M PB pH 7.4 for 4 h and cryopreserved in serial dilutions of 10–30% sucrose at 4 °C for at least 24 h, embedding using O.C.T. compound, retinas were sliced transversely (12 μm) using a cryostat (Leica CM1860 UV, # 76404-216) and stored at –20 °C until the assay. For in vitro experiments, the cells were fixed in PFA 4% for 1 h at 4 °C under constant agitation. Once fixed, the samples were washed twice using cold 0.9% phosphate-buffered saline (PBS) for 5 min each, and then incubated in 0.9% PBS + 0.4% Triton X-100 at room temperature (RT) for 40 min to permeabilize the cells. After incubation, the cells were washed in 0.9% PBS with 0.1% Tween 20 for 5 minutes and then incubated with a 1% albumin-blocking solution for 30 minutes.

Retinal cells and sections were incubated overnight at room temperature (RT) with the primary antibody in a solution containing 5% normal donkey serum and 0.5% Triton X-100 in phosphate-buffered saline (PBS) at 4 °C with agitation (for cells) or at RT (for tissue). On the following day, the fluorescence-coupled secondary antibody Alexa 488 and/or 546 (1:500–1:3000, Invitrogen), and 4’6-Diamidino-2-phenylindole dihydrochloride (Nuclei, DAPI, 1:60.000) diluted in 0.5% Triton X-100 and 0.1 M PB for 2 h (for tissue) and 3 h (for cells) at RT. All antibodies and specific concentrations used in this study are listed in Supplementary Table S1. Experimental controls were prepared by omitting the primary antibodies. For in vivo experiments, sections were mounted using VectaShield (Vector Labs, Burlingame, CA, USA). Cells were photographed under Nikon TS100F (Nikon Instruments Inc., Melville, NY, USA). The retinal sections were photographed using a DM 5500 microscope (Leica Microsystems, Germany) equipped with a camera for image capture (DFC 365 FX, Leica Microsystems, Germany). Figures were prepared using Adobe Photoshop CC 2014 (Adobe Systems Inc., San Jose, CA, USA). Manipulation of the images was limited to adjustments of brightness and contrast for the entire image.

Cell counting was utilized both in vitro and in vivo. For in vitro analysis, the positive cells were manually counted using the ImageJ cell counter plugin. The number of cells was normalized to the total number of DAPI+ cells in the same region. Microglial and astrocytic branching and morphology parameters were quantified as previously reported [73]. For Fractal and Skeleton, the ‘*n*’ considered was for each isolated microglia, and for astrocytes, the skeleton, the ‘*n’* considered was for micrography. The neuron neurite analysis was utilized for in vitro data analysis only. The plugin NeuroJ of the software ImageJ was used. Each neuron was isolated and quantified; “n” was the number of individual neurons per well/condition, 20–35 neurons per well, for each biological replicate. All images analyzed were acquired with the same exposure intensity.

### Whole mount

For whole-mount experiments, the animals were anesthetized and euthanized. After decapitation, the eyes were dissected and post-fixed in 4% PFA in 0.1 M PB pH 7.4 for 2 h. The eyes were washed in PBS, and the retinas were carefully collected and washed with PBS. The retinas were incubated with the primary antibody in a solution containing 5% normal donkey serum and 0.5% Triton X-100 in PBS at 4 °C in agitation. On the following day, the fluorescence-coupled secondary antibody Alexa 488 and/or 546 (1:500–1:3000, Invitrogen), and 4’6-Diamidino-2-phenylindole dihydrochloride (Nuclei, DAPI, 1:60.000) diluted in 0.5% Triton X-100 and 0.1 M PB for 2 h at RT. All antibodies and specific concentrations used in this study are listed in Supplementary Table S1. Z-stack images were acquired using a DM 5500 microscope (Leica Microsystems, Germany), coupled to a camera for image capture (DFC 365 FX, Leica Microsystems, Germany). The 2D reconstructions were created using ImageJ stacking and Z-projection. Figures were prepared using Adobe Photoshop CC 2014 (Adobe Systems Inc., San Jose, CA, USA).

### Hematoxylin-eosin stain (HE)

Fixed retinas were cryosectioned and rehydrated with xylene and serial decreasing ethanol concentrations (100%, 95%, 80%, 5 min each). Before the hematoxylin stain, the sections were washed with deionized water for 7 min, stained with a hematoxylin solution for 2 min, and then washed with water for an additional 7 min. Then, sections were stained with an eosin solution for 5 min, post-dehydrated, and washed with xylol. The slices were dried, then sealed with coverslips and DPX (Sigma-Aldrich, #06522). The retinal sections were analyzed under a bright-field microscope (DM5500, Leica Microsystems, Germany) coupled to a camera for image capture (DFC365FX, Leica Microsystems, Germany). The areas of specific retinal layers were measured using ImageJ.

### Terminal deoxynucleotidyl transferase (TdT)-mediated dUTP nick-end labeling (TUNEL) assay

Briefly, TUNEL assay (Life Technologies, Cat #C10618) was performed in retinal sections (12 μm) assembled on gelatinized slides [30]. The fixed tissue was washed twice for 10 minutes in 0.05 M phosphate-buffered saline (PBS). After drying, the slides were incubated in a 0.1% sodium citrate solution for 2 min at 4 °C. Following incubation, the slides were washed twice for 5 min in 0.1 M phosphate-buffered saline (PBS) and then dried. TdT Reaction Buffer was added for 10 min, followed by the TdT reaction solution for 1 h at 37 °C, and the cells were washed in PBS containing 3% BSA and 0.1% Triton X-100 for 5 min. The slides were then incubated for 1 h with the Click-iT Plus TUNEL solution prepared according to the manufacturer’s protocol. The slides were rewashed in 3% BSA and 0.1% Triton X-100. After the assay steps, the tissues were washed with PBS 0.9% and incubated with DAPI for 20 min. After, the slides were washed with 0.1 M PB, dried and closed with coverslips.

### Multiplex magnetic bead array

Vehicle- and 2-APB-treated retinas were harvested 4 days after the subretinal injection and homogenized in an EDTA-free Protease Inhibitor Cocktail (Roche, #11836170001). Homogenates were centrifuged for 20 min at 14,000 × *g*, 4 °C. BCA method was used to determine protein concentration, and bovine serum albumin was used as the standard. The following steps were performed according to the manufacturer’s instructions using the commercial kit (Millipore, #MCYTOMAG-70K), and the cytokines quantified simultaneously were IL-1β, IL-4, IL-6, IL-10, IL-13, IL-17, IFN-y, TNF-α, VEGF, and GCSF. Cytokine values were normalized by the total protein concentration of each sample. Th1 and Th2 cytokines were divided according to the literature. Th1/Th2 dominance was determined by the INFy/IL-4 ratio. The mean of Pearson’s correlation was obtained in the correlation matrix report of heatmap statistical analysis using NCSS software (NCSS, LLC, Kaysville, Utah, USA, ncss.com/software/ncss.).

### Hierarchical clustering analysis (HCA)

We combined hierarchical clustering analysis (HCA) with dimensionality reduction to characterize microglial morphological changes after 2-APB treatment. Skeleton and fractal descriptors for each cell were preprocessed by removing low-variance features and those with Pearson |*r*| ≥ 0.95. Data from each experimental group were then normalized via StandardScaler and merged. We applied UMAP to project cells into two dimensions, visualized them in scatter plots colored by treatment, and overlaid group centroids to highlight treatment-induced shifts. The optimal cluster number was chosen by the elbow method and confirmed by visual inspection of dendrograms. HCA with Ward’s linkage assigned cells to clusters based on a distance threshold. We calculated each cluster’s relative frequency (cells per cluster/total cells) and generated a heatmap of z-normalized feature means to compare morphological profiles across clusters. All steps were implemented in Python 3.9 using pandas, numpy, scikit-learn, scipy, matplotlib, seaborn, and umap-learn.

### Statistical analysis

Sample sizes were chosen based on prior studies using similar experimental paradigms and effect sizes, as well as on empirical variability observed in pilot experiments, rather than on a formal a priori power calculation. Post-hoc tests were selected based on the sample’s characteristics. For in vivo experiments, treatments and vehicles were assigned to contralateral eyes, which served as internal controls. For in vitro experiments, samples were processed in parallel without formal randomization. Data were reported as mean differences with standard errors of the mean. Qualitative analyses, such as morphological descriptions, were performed through blind evaluation whenever possible. Investigators were not blinded to group allocation during experiments or data analysis. To assess data normality, the D’Agostino & Pearson test was used. Depending on the outcome of this test, different statistical methods were used: if the data passed the normality test, parametric tests were applied; if the data did not pass the normality test, non-parametric tests were used. The text and figure captions indicate the specific statistical tests and the ‘n’ of the sample used for analysis. All comparisons were performed as described in the figure legends. A *p-value* < 0.05 was considered statistically significant. The statistical analysis was performed using GraphPad Prism version 6.00 for Windows (GraphPad Software, La Jolla, California, USA). Outliers were removed with GraphPad Prism “identify outliers”, ROAT test with aggressiveness set to 1%.

## Supplementary information

Supplementary Figure 1

Supplementary Figure 2

Supplementary Figure 3

Supplementary Figure 4

Supplementary Material

Supplementary Tables

## Data Availability

The datasets generated and/or analyzed during the current study are available from the corresponding author upon reasonable request.
