# Supplementary figures and images for: Store-operated calcium entry drives alcohol-exacerbated neuroinflammation in retinal degeneration
Source: Cell Death Discov. 2026 Mar 31;12:222. doi: 10.1038/s41420-026-03074-2 (PMC13184073; doi:10.1038/s41420-026-03074-2)

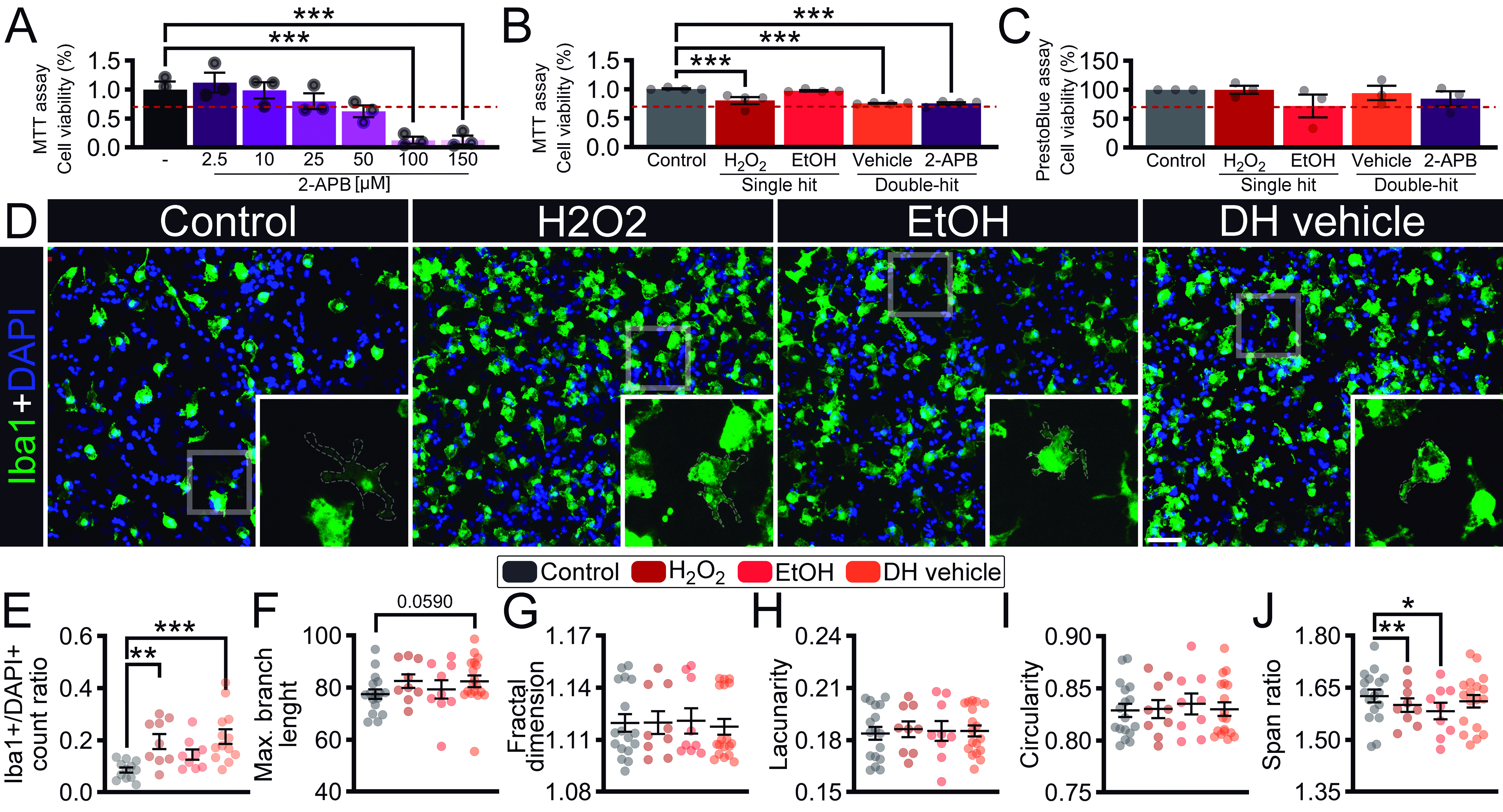

Supplement: Supplementary file 1 — Supplementary Figure 1 [file 41420_2026_3074_MOESM1_ESM.jpg]

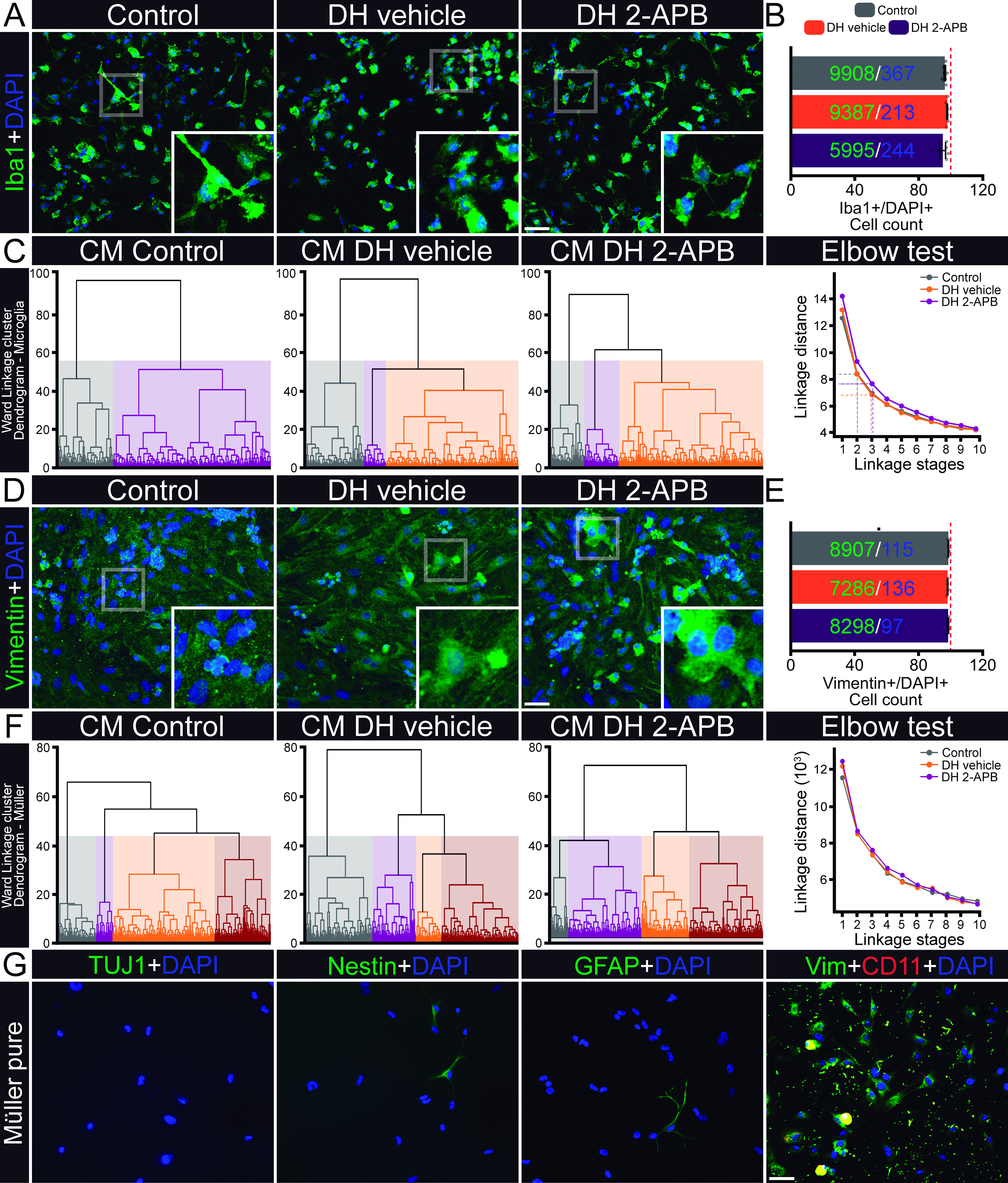

Supplement: Supplementary file 2 — Supplementary Figure 2 [file 41420_2026_3074_MOESM2_ESM.jpg]

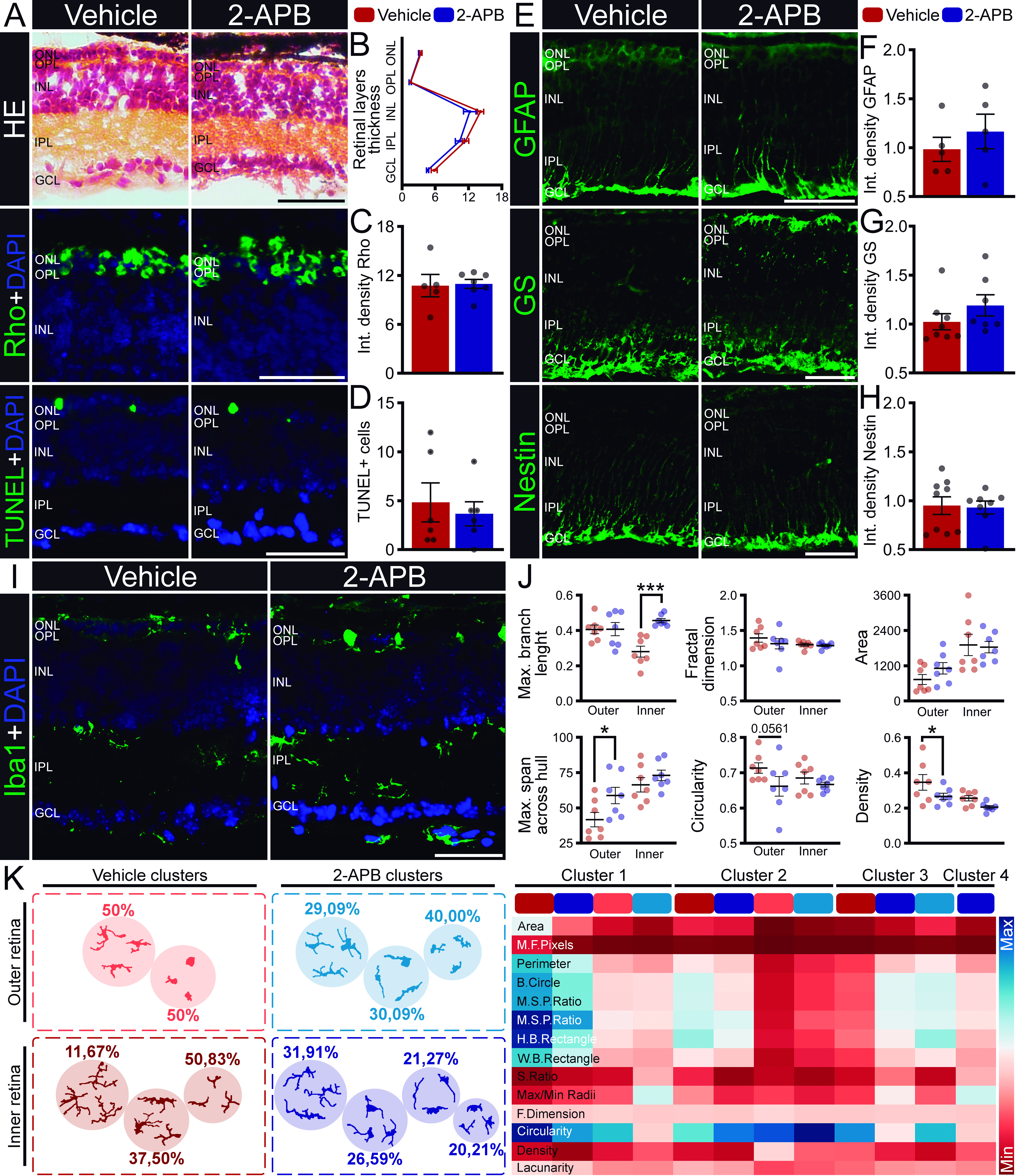

Supplement: Supplementary file 3 — Supplementary Figure 3 [file 41420_2026_3074_MOESM3_ESM.jpg]

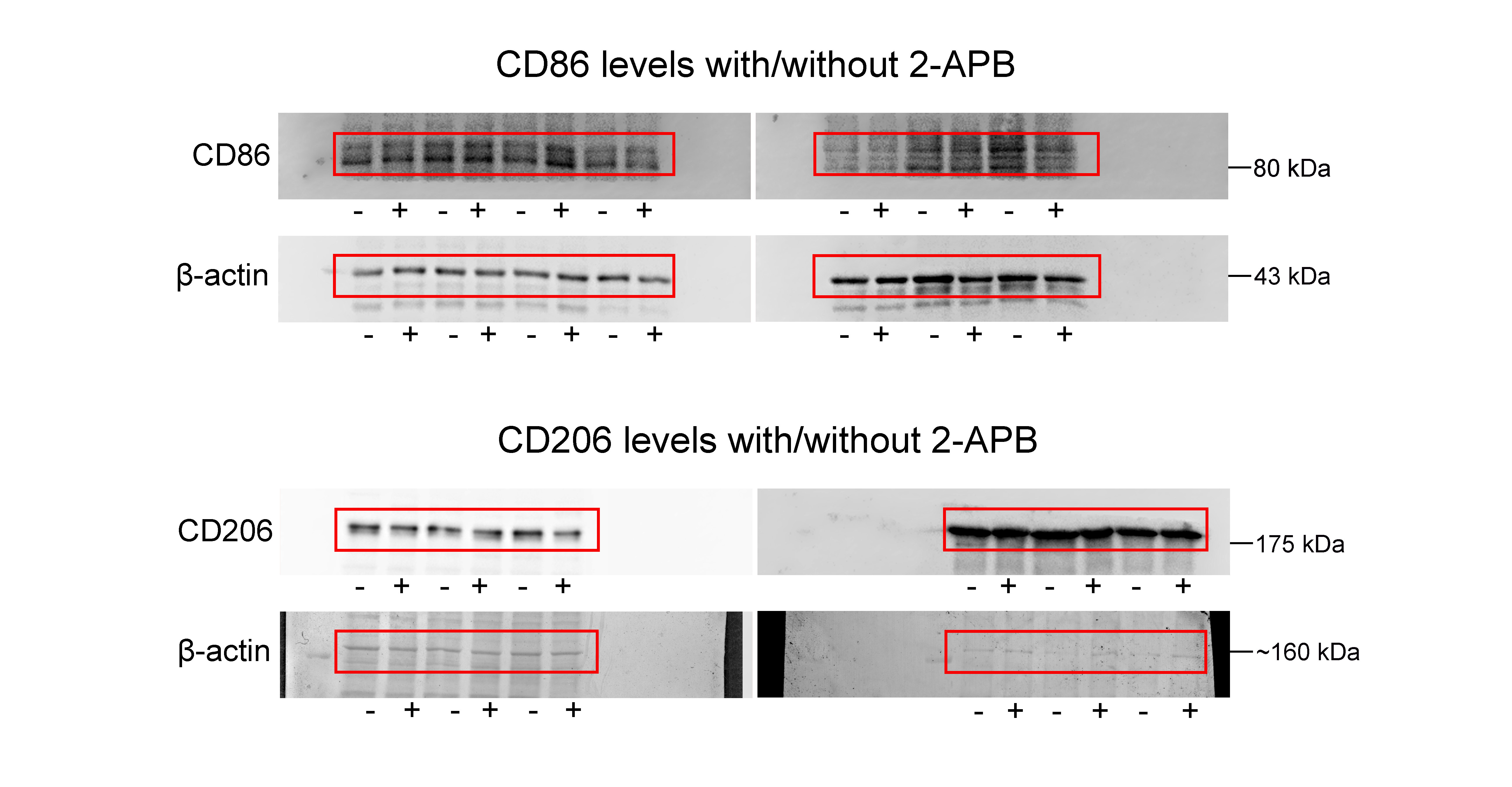

Supplement: Supplementary file 4 — Supplementary Figure 4 [file 41420_2026_3074_MOESM4_ESM.jpg]
