# Supplementary material for: Store-operated calcium entry drives alcohol-exacerbated neuroinflammation in retinal degeneration
Source: Cell Death Discov. 2026 Mar 31;12:222. doi: 10.1038/s41420-026-03074-2 (PMC13184073; doi:10.1038/s41420-026-03074-2)
Supplement: Supplementary file 5 — Supplementary Material [file 41420_2026_3074_MOESM5_ESM.docx]

# SUPPLEMENTARY FIGURES

Supplementary Figure 1. In vitro double‑hit model in primary retinal cells: effects of 2‑APB on cellular metabolism and microglial inflammatory response. (A) The viability of mixed primary cell cultures treated with a vehicle (black, considered as 100% viability) or different dosages of 2-APB, with a red line marking 70% cell viability as evaluated by the MTT assay. (B) The viability of mixed primary cell cultures under single and double hit pre-treatment with or without 2-APB, with a red line marking 70% cell viability as evaluated by the MTT assay. (C) The viability of mixed primary cell cultures under single and double hit pre-treatment with or without 2-APB, with a red line marking 70% cell viability as evaluated by the PB assay. (D) Representative immunofluorescence of the ionized calcium-binding adapter molecule 1 (Iba1, green – microglia), counterstained with 4′,6-diamidino-2-phenylindole (DAPI, blue – nucleus), with digital magnification of microglia exposed to different conditions: control, 50 µM H2O2, EtOH 50 mM, and DH pre-treated with vehicle, respectively. (E) Ratio of the number of Iba1-positive cells per DAPI-labeled nuclei, n = 75-90 images from 10-13 wells. (F-J) Analysis of morphological parameters of microglia isolated from each group, considering (F) maximum branch length, (G) fractal dimension, (H) lacunarity, (I) circularity, (J) span ratio, n = 240-350 individual microglia. (A-J) Experiments n = 3-4 biological replicates × 3-6 technical replicates. Values indicate mean ± SEM. (A-C) Bars represent the standard errors of means. (E-J) The scatter dot plot represents mean ± SEM. The values that compose the mean are expressed as black dots in both graphs, *P < 0.05, ***P < 0.001, ****P < 0.0001, in Mixed-effects followed by Two-stage step-up method of Benjamini, Krieger and Yekutieli post-hoc. Scale bar: 50 μm.

Supplementary Figure 2 – Cell culture purity, elbow tests, and other data. (A) Representative immunofluorescence of ionized calcium-binding adapter molecule 1 (Iba1, green – microglia), counterstained with 4′,6-diamidino-2-phenylindole (DAPI, blue – nucleus), displaying digital magnification of microglia exposed to different conditions: control, DH pre-treated with vehicle, and DH pre-treated with 2-APB. (B) Ratio of the number of Iba1-positive cells per DAPI-labeled nuclei, n = 6 wells. (C) Hierarchical clustering dendrogram using Ward linkage of microglial cells sampled from each group, with the abscissa representing individual microglia and the ordinate corresponding to the linkage distance measured based on 21 features, defining 2-3 distinct morphological clusters as determined by the elbow method following Thorndike's procedure. (D) Representative immunofluorescence of vimentin (green – Müller), counterstained with 4′,6-diamidino-2-phenylindole (DAPI, blue – nucleus), showing digital magnification of microglia exposed to different conditions: control, DH pre-treated with vehicle, and DH pre-treated with 2-APB. (E) Ratio of the number of vimentin-positive cells per DAPI-labeled nuclei, n = 4 wells. (F) Hierarchical clustering dendrogram using Ward linkage of microglial cells sampled from each group, where the abscissa represents individual microglia and the ordinate corresponds to the linkage distance measured based on 21 features, defining 2-3 distinct morphological clusters determined by the elbow method following Thorndike's procedure. Experiments n = 3-4 biological replicates × 3-4 technical replicates. Values indicate mean ± SEM. (B, E) Bars represent standard errors of means. The values that compose the mean are expressed as black dots in both graphs.

Supplementary Figure 3. 2‑APB treatment modulates neuroinflammatory responses in the arRP animal model. (A) Representative photomicrographs of hematoxylin and eosin staining (HE), immunofluorescence of rhodopsin (Rho, green – rod photoreceptor cells), and TUNEL assay (green, indicating cell death), counterstained with 4′,6-diamidino-2-phenylindole (DAPI, blue – nucleus), for vehicle and 2-APB treatments injected via subretinal injection in C3H/HeJrd1 retinas 13 days after birth, collected at 17 days of age. (B) Retinal thickness per layer. (C) Integrated density of Rho, n = 6. (D) TUNEL-positive cell count, n = 6. (E) Representative immunofluorescence of glial fibrillary acidic protein (GFAP, green – macroglias), glutamine synthetase (GS, green - microglia), and nestin (green – macroglia), counterstained with DAPI, for vehicle and 2-APB treatments. (F) Integrated density of GFAP, n = 5. (G) Integrated density of GS, n = 7. (H) Integrated density of nestin, n = 9. (I) Representative immunofluorescence Iba1, counterstained with DAPI, for vehicle and 2-APB treatments. (J) Analysis of morphological parameters of microglia isolated from Vehicle- and 2-APB-injected retinas, considering maximum branch length, fractal dimension, area, maximum span across the hull, circularity, and density, separated into inner (INL-GCL) and outer (OPL-ONL) retina, n = 50-120 individual microglia,. (K) Representative cells from 2-4 distinct morphological clusters defined by a hierarchical clustering dendrogram using Ward linkage of microglial cells sampled from each group. Values indicate mean ± SEM. Bars represent standard errors of the means. The scatter dot plot represents mean ± SEM, with the values composing the average expressed in black dots. (C-H) Unpaired Student's t-test, (B) two-way ANOVA followed by Bonferroni’s post-hoc. (J) The scatter dot plot represents mean ± SEM. *P < 0.05, **P < 0.01, ***P < 0.001, in (C) two-way ANOVA followed by Bonferroni’s post-hoc. The layers indicate the approximate localization of ONL: outer nuclear layer, OPL: outer plexiform layer, IPL: inner plexiform layer, INL: inner nuclear layer, GCL: ganglion cell layer. Scale bar: 50 μm.

Supplementary Figure 4. Uncropped western blotting. (-) represents vehicle and (+) 2-APB injected retinas.

**Supplementary Table S1.** List of antibodies and dilutions.

**Supplementary Table S2.** Microglial cell count and morphological parameters from fractal and skeleton analysis comparing single and double-hit to control. Statistical analyses were performed using a mixed-effects model followed by the Benjamini, Krieger, and Yekutieli two-stage linear step-up procedure (post-hoc test). Cell count was analyzed by one-way ANOVA followed by Tukey’s multiple comparisons test. N = 9-18 technical replicates.

**Supplementary Table S3.** Microglial cell count, and morphological parameters from fractal and skeleton analysis comparing double-hit (Vehicle & 2-APB) to control. Statistical analyses were performed using a mixed-effects model followed by the Benjamini, Krieger, and Yekutieli two-stage linear step-up procedure (post-hoc test). Cell count was analyzed by one-way ANOVA followed by Tukey’s multiple comparisons test. N = 17-18 technical replicates.

**Supplementary Table S4.** Astrocyte cell count and morphological parameters from skeleton analysis comparing double-hit (Vehicle & 2-APB) to control. Statistical analyses were performed using a mixed-effects model followed by the Benjamini, Krieger, and Yekutieli two-stage linear step-up procedure (post-hoc test). Cell count was analyzed by one-way ANOVA followed by Tukey’s multiple comparisons test. n = 3-6 technical replicates.

**Supplementary Table S5 -** Neurite data analysis comparing double-hit to control. Statistical analyses were performed using a mixed-effects model followed by the Benjamini, Krieger, and Yekutieli two-stage linear step-up procedure (post-hoc test). n = 3-6 technical replicates.

**Supplementary Table S6.** Microglial cell count and morphological parameters from fractal and skeleton analyses comparing treatments with conditioned medium from pure microglia. Statistical analyses were performed using a mixed-effects model followed by the Benjamini, Krieger, and Yekutieli two-stage linear step-up procedure (post-hoc test). Cell count was analyzed by one-way ANOVA followed by Tukey’s multiple comparisons test. N = 6 technical replicates.

**Supplementary Table S7.** Microglial cell count and morphological parameters from fractal and skeleton analyses comparing treatments with conditioned medium from pure Müller cells. Statistical analyses were performed using a mixed-effects model followed by the Benjamini, Krieger, and Yekutieli two-stage linear step-up procedure (post-hoc test). Cell count was analyzed by one-way ANOVA followed by Tukey’s multiple comparisons test. N = 5-6 technical replicates.

**Supplementary Table S8.** Microglial cell count, and morphological parameters from fractal and skeleton analysis comparing microglia from vehicle and 2-APB injected retinas (Vertical slice). Statistical analyses were performed using a mixed-effects model followed by the Benjamini, Krieger, and Yekutieli two-stage linear step-up procedure (post-hoc test). N = 7 animals.

**Supplementary Table 9.** Microglia cell count, and morphological parameters from fractal and skeleton analysis comparing microglia from vehicle and 2-APB injected retinas (Whole mount). Statistical analyses were performed using a mixed-effects model followed by the Benjamini, Krieger, and Yekutieli two-stage linear step-up procedure (post-hoc test). N = 7 animals.

**Supplementary Table 10.** GFAP mean intensity, and morphological parameters from skeleton analysis comparing GFAP from vehicle and 2-APB injected retinas (Whole mount). Statistical analyses were performed using a paired Student’s t-test. Mean intensity values were analyzed by two-way ANOVA followed by the Benjamini, Krieger, and Yekutieli two-stage linear step-up procedure (post-hoc test). n = 6 animals.
